# Simultaneous Aerosol and Intramuscular Immunization with Influenza Vaccine Induces Powerful Protective Local T Cell and Systemic Antibody Immune Responses in Pigs

**DOI:** 10.4049/jimmunol.2001086

**Published:** 2020-12-16

**Authors:** Veronica Martini, Basu Paudyal, Tiphany Chrun, Adam McNee, Matthew Edmans, Emmanuel Atangana Maze, Beckie Clark, Alejandro Nunez, Garry Dolton, Andrew Sewell, Peter Beverley, Ronan MacLoughlin, Alain Townsend, Elma Tchilian

**Affiliations:** * The Pirbright Institute, Pirbright GU24 0NF, United Kingdom;; † Weatherall Institute of Molecular Medicine, University of Oxford, Oxford OX3 9DS, United Kingdom;; ‡ UK Animal and Plant Health Agency-Weybridge, New Haw, Addlestone KT15 3NB, United Kingdom;; § Division of Infection and Immunity, Cardiff University School of Medicine, Cardiff CF14 4XN, United Kingdom;; ¶ National Heart and Lung Institute, Imperial College London, London W2 1PG, United Kingdom; and; ‖ Aerogen Ltd., Dangan, Galway H91 HE94, Ireland

## Abstract

A vaccine providing both powerful Ab and cross-reactive T cell immune responses against influenza viruses would be beneficial for both humans and pigs. In this study, we evaluated i.m., aerosol (Aer), and simultaneous systemic and respiratory immunization (SIM) by both routes in Babraham pigs, using the single cycle candidate influenza vaccine S-FLU. After prime and boost immunization, pigs were challenged with H1N1pdm09 virus. i.m.-immunized pigs generated a high titer of neutralizing Abs but poor T cell responses, whereas Aer induced powerful respiratory tract T cell responses but a low titer of Abs. SIM pigs combined high Ab titers and strong local T cell responses. SIM showed the most complete suppression of virus shedding and the greatest improvement in pathology. We conclude that SIM regimes for immunization against respiratory pathogens warrant further study.

## Key Points

- Simultaneous aerosol and i.m. immunization reduces viral load and pathology.
- Simultaneous immunization induces strong protective local and systemic responses.
- The hierarchy of NP-specific T cells in blood does not reflect local lung responses.

## Introduction

Immunization against infectious diseases has been practiced for several centuries, but identifying the best method of administering a vaccine is still often a matter of empirical experimentation. Three major considerations should make rational immunization easier. The first is the importance of pathogen-associated molecular patterns, which are essential for triggering an immune response. The second is that the site of immunization programs lymphocytes to return to it. The third is that local immune responses are critical for protection against mucosal infection and that many lymphocytes reside in nonlymphoid tissues and provide tissue-resident memory. Immunization at the site of infection offers the advantage that an immune response is generated at the site of entry of the pathogen and should provide immediate protection.

Immunization of the respiratory tract has been demonstrated to be highly effective against influenza, and cold-adapted, live attenuated influenza vaccine (LAIV) has efficacy rates of 75–80% in children and additionally gives some cross-reactive protection against antigenically distinct strains. However, LAIV is not so effective in adults or the elderly. In contrast, the traditional i.m.-inactivated seasonal human influenza vaccine provides 10–60% efficacy and induces strain-specific immunity by generation of subtype specific Abs so that repeated annual vaccination to match new influenza variants is required (1–3). Therefore, there is an urgent need for new immunization strategies for influenza that provide broad and long-lasting protection.

One such strategy, which has been explored against tuberculosis (Tb), is to combine the advantages of local and systemic immunization. Parenteral Bacillus Calmette–Guérin (BCG) priming followed by intranasal boosting with an adenovirus-vectored vaccine expressing Ag 85A (Ad85A) markedly enhanced protection in mice (4). We have shown that simultaneous systemic and respiratory immunization (SIM) with BCG in mice or BCG/BCG and BCG/Ad85A in cattle enhanced protection against Tb challenge (5, 6). Uddback et al. (7, 8) have used this strategy with an adeno vector expressing influenza nucleoprotein (NP) and shown greatly improved and durable protection against heterosubtypic influenza challenge in mice. These data prompted us to test SIM in the pig model using the candidate broadly protective signal minus influenza vaccine S-FLU. S-FLU is a pseudotyped influenza virus, lacking the hemagglutinin (HA) signal sequence and therefore limited to a single cycle of replication. S-FLU induces a strong cross-reactive T cell response but a minimal humoral response to HA when administered mucosally (9, 10). We have shown that aerosol (Aer) delivery of S-FLU reduces lung viral load when partially matched to the challenge virus, correlating with a local lung T cell immune response (11). When S-FLU was completely mismatched to the challenge virus, pathology but not viral load was reduced. This suggests that in the absence of an Ab response, lung T cell immunity can reduce disease severity (12). By contrast, the same S-FLU preparation induced sterile immunity to the matched challenge virus and reduced replication and Aer transmission to naive recipients following mismatched viral challenge in ferrets (12). The pig is a more relevant large animal model because it is a natural host for influenza viruses and has very similar respiratory anatomy to humans (13, 14). Pigs and humans are infected by the same subtypes of influenza A viruses and are integrally connected in the ecology of influenza.

In this study, we evaluated the efficacy of SIM with S-FLU against H1N1pdm09 challenge using inbred Babraham pigs, allowing a more refined analysis of the specificity of the immune responses using MHC class I tetramers to previously defined immunodominant NP epitopes (15).

## Materials and Methods

### Vaccine and virus challenge

The H1N1 signal minus influenza vaccine (S-FLU) [eGFP*/N1(A/Eng/195/2009)] H1(A/Eng/195/2009) containing the internal genes of A/Puerto Rico/8/1934 virus was produced as previously described (9). The swine isolate H1N1 A/swine/England/1353/2009 (H1N1pdm09) was used to infect the pigs.

### Animal immunization and challenge study

The animal experiment was approved by the ethical review process at Animal and Plant Health Agency and followed the U.K. Government Animal (Scientific Procedures) Act 1986. Twenty-four 5–6-wk-old Babraham large, white, inbred female and male pigs were randomized into four groups of six animals as follows: 1) the first group received S-FLU by Aer as previously described (12); 2) the second group was immunized i.m. with S-FLU (i.m.); 3) the third group was immunized simultaneously i.m. and by Aer with S-FLU (SIM), and 4) the fourth group was an unimmunized control group. Two pigs reached their humane end points because of a pre-existing heart condition, limiting the number of pigs in the control and i.m. groups to five animals. During immunization, all the animals were sedated with a mixture of 4.4 mg/kg Zoletil (zolazepam) (Virbac) and 0.044 mg/kg Domitor (m*edetomidine*) (Orion Pharma). Aer immunization was performed using a small droplet size, vibrating mesh nebulizer (Aerogen Solo; Aerogen) attached to a custom-made veterinary mask (12). For Aer immunization, 2 ml of S-FLU containing 7 × 10^7^ 50% tissue culture infective dose (TCID_50_) S-FLU was administered over 6–10 min. For i.m. administration, the vaccine stock was diluted to a final volume of 4 ml containing 7 × 10^7^ TCID_50_, and 2 ml was administered to each trapezius muscle behind the ear. Pigs in the SIM group received 2 ml of 3.5 × 10^7^ TCID_50_ S-FLU by Aer (as described above) and 3.5 × 10^7^ TCID_50_ S-FLU delivered in 4 ml i.m. (2 × 2 ml in each trapezius muscle). The animals were boosted 3 wk later in a similar manner. Three weeks after the boost, all groups were challenged with 2.8 × 10^6^ PFU of H1N1pdm09 intranasally using a mucosal atomization device (MAD300; Wolfe Tory Medical). For logistic reasons, the challenge was performed in two different days so that half of the animal in each group were challenged on 23 d postboost (DPB) and the remaining half on 25 DPB. Animals were humanely culled at 4 d postchallenge (DPC) with an overdose of pentobarbital sodium anesthetic. At the second cull, 1 mg/kg of anti-CD3 purified mAb (PPT3 clone, produced in-house) was infused i.v. to the pigs 10 min prior to sacrifice. Because no difference was found in analyses of the samples challenged on different days, the results are presented together. Gross and histopathological analyses were performed as previously described (11, 12, 16). Briefly, the lungs were removed, and digital photographs were taken of the dorsal and ventral aspects. Macroscopic pathology was scored blind as previously reported (17). The percentage of the lung displaying gross lesions for each animal was calculated using image analysis software (Fiji ImageJ) on the digital photographs. Lung tissue samples from cranial, middle, and caudal lung lobes were taken from the right lung and collected into 10% neutral-buffered formalin for routine histological processing. Formalin-fixed tissues were paraffin wax embedded, and 4-μm sections were cut and routinely stained with H&E. Immunohistochemical detection of influenza A virus NP was performed in 4-μm tissue sections as previously described (18). Histopathological changes in the stained lung tissue sections were scored by a veterinary pathologist blinded to the treatment group. Lung histopathology was scored using five parameters (necrosis of the bronchiolar epithelium, airway inflammation, perivascular/bronchiolar cuffing, alveolar exudates, and septal inflammation) on a five-point scale of 0–4 and then summed to give a total slide score ranging from 0–20 per lobe (∼1.5 × 1.5 cm^2^) and a total animal score from 0 to 60 (11). A mean score for the three lung lobes was calculated for each animal. The individual lung lobes were also scored using the “Iowa” method, which takes into account the amount of viral Ag present in the sample, as described (19).

### Tissue sample processing

Blood, spleen, bronchoalveolar lavage (BAL), and lung lobes were processed as described previously (11, 12). Trachea and nasal turbinate mucosae were separated from cartilage with tweezers and digested for 2 h at 37°C in RPMI 1640 supplemented with 100 U/ml penicillin, 100 mg/ml streptomycin, 2 mM l-glutamine (all from Life Technologies), 2 mg/ml collagenase D (Roche), 1 mg/ml dispase, and 1 mg/ml of DNase (both from Sigma-Aldrich). Tissues filaments were then mashed with the plunger of a syringe. Isolated cells were then passed through a 70-μm cell strainer and RBC lysed before cryopreservation in FCS and 10% DMSO. Nasal swabs (one per nostril) were taken daily following infection with H1N1pdm09. Viral titer in nasal swabs and BAL was determined by plaque assay on MDCK cells as previously described (11).

### Serological assays

ELISA was performed using recombinant HA (from A/England/195/2009) containing a C-terminal thrombin cleavage site, a trimerization sequence, a hexahistidine tag, and a BirA recognition sequence as previously described (20). Microneutralization (MN) was performed using standard procedures as described previously (9, 16).

### Enzyme-linked lectin assay

Enzyme-linked lectin assay (ELLA) was used to quantify neutralization of neuraminidase (NA) enzymatic activity by Ab as described before (21). Briefly, NUNC Immuno 96-microwell plates (Sigma-Aldrich) were coated overnight at 4°C with 25 μg/ml fetuin (Sigma-Aldrich) in PBS containing 0.02% sodium azide. Heat-inactivated sera and BAL were serially diluted in DMEM supplemented with 0.1% BSA, 100 U/ml penicillin, 100 mg/ml streptomycin, and 2 mM l-glutamine starting at 1:40 and 1:4, respectively. H7N1 S-FLU [eGFP/N1(A/Eng/09)] H7(Netherlands/219/2003) was used to minimize any potential steric effect of Abs binding to H1 HA. An optimal concentration of H7N1 S-FLU was added to the diluted Abs for 20 min on a plate shaker. One hundred microliters of the mixture of virus and diluted samples were then transferred to the washed coated plate and incubated for 18 h at 37°C. Peanut agglutinin–conjugated with HRP (Sigma-Aldrich) was added at 1 μg/ml in PBS and incubated at room temperature for 2 h. The plates were washed and developed with 50 μl of TMB (BioLegend); after 5 min, the reaction was stopped with 50 μl of 1 M sulfuric acid and absorbance measured at 450 and 630 nm. The 50% inhibition titer was calculated as the highest dilution above the IC_50_ line (midpoint between the signal generated by virus-only and medium-only wells).

### B cell ELISpot assay

Cryopreserved lymphocytes from blood, spleen, and tracheobronchial lymph nodes (TBLN) were used. Then, 10^7^ cells/well were stimulated in each well of a 12-well plate with the TLR7 agonist R484 at 1 μg/ml in RPMI 1640 supplemented with 100 U/ml penicillin, 100 mg/ml streptomycin, 10% FBS, and 0.1% 2-Mercaptoethanol (all from Life Technologies). After 48 h, cells were washed twice with medium and counted. Then, 5 × 10^5^ cells were distributed in duplicate in assay plates for the detection of HA-specific Ab-secreting cells (ASC) and in negative control wells, whereas 0.5 × 10^5^ cells per well were plated to detect all Ig-secreting cells (positive controls). Assay plates were MultiScreen-HA ELISpot plates (Millipore), coated with anti-porcine IgG, clone MT421 (Mabtech), or anti-porcine IgA, A100–102A (Bethyl Laboratories) 1/500 in carbonate buffer overnight at 4°C. After overnight incubation at 37°C, the plates were washed five times with PBS containing 0.05% Tween 20 and incubated with biotinylated HA for detection of HA-specific B cells (obtained as described before), biotinylated keyhole limpet hemocyanin (Sigma-Aldrich) as a negative control, both at 0.1 μg/ml in PBS, biotinylated anti-porcine IgG (MT424; Mabtech), or anti-porcine IgA (A100-102-B; Bethyl Laboratories) at 1/1000 in PBS to detect all Ig-secreting cells. After a 2-h incubation, plates were washed, and streptavidin alkaline phosphatase (Invitrogen) was added for another hour. The plates were then developed and read. Spots detected with keyhole limpet hemocyanin were subtracted from the HA response, and data were presented as ASC per million cells.

### Flow cytometry

Cryopreserved lymphocytes from BAL were thawed and stimulated with H1N1pdm09 (multiplicity of infection 1) or medium as a control for 18 h at 37°C prior to GolgiPlug (BD Biosciences) addition as per manufacturer instructions. Following a 5-h incubation with GolgiPlug at 37°C, cells were stained with surface markers (Table I) before fixation and permeabilization using Cytofix Cytoperm (BD Biosciences). Intracellular staining was then performed, and the samples were analyzed using an LSRFortessa (BD Biosciences). Data were analyzed by Boolean gating using FlowJo v10 (Tree Star). For identification of tissue-resident memory T cells (TRM), three animals from each vaccinated group and two control animals were infused i.v. with 1 mg/kg of purified CD3 mAb (clone PPT3) and sacrificed 10 min later, as described above. Cryopreserved lymphocytes isolated from the different tissues were labeled with anti-mouse IgG1-allophycocyanin, which labels the circulating intravascular cells, for 20 min at 4°C. After two washes with PBS, normal mouse serum was added to block any remaining binding sites of the secondary Ab. The lymphocytes were then stained with surface markers (Table I), including anti-porcine CD3-FITC (clone PPT3; Bio-Rad Laboratories). As not all CD3 sites would be saturated by i.v. anti-CD3 mAb, circulating T cells are double labeled, whereas tissue-resident T cells are positive only for the ex vivo anti–CD3-FITC.

NP tetramer staining was performed on cryopreserved lymphocytes from PBMC, lung, BAL, trachea, and nasal turbinate as previously described (15). Briefly, biotinylated NP peptide-loaded swine leukocyte Ag monomers were freshly assembled into tetramer with streptavidin Brilliant Violet (BV) 421 or BV650 (both from BioLegend). Two million mononuclear cells were incubated with protease kinase inhibitor in PBS for 30 min at 37°C, and tetramers were added to the cells on ice for another 30 min. Surface staining with optimal Abs concentration in FACS buffer (PBS supplemented with 2% FCS and 0.05% sodium azide) was performed on ice for 20 min (Table I). Samples were washed twice with FACS buffer and fixed in 1% paraformaldehyde before analysis using an LSRFortessa (BD Biosciences).

### Statistical analysis

GraphPad version 8.4.1 was used for statistical analysis. Kruskal–Wallis test was used for the comparison between groups of viral load, pathology, Ab, and T cells responses. Two-way ANOVA was used for the comparison of neutralizing Ab and to analyze the hierarchy of the response in the different tissues within the same group.

## Results

### Virus load and lung pathology

**FIGURE 1.**
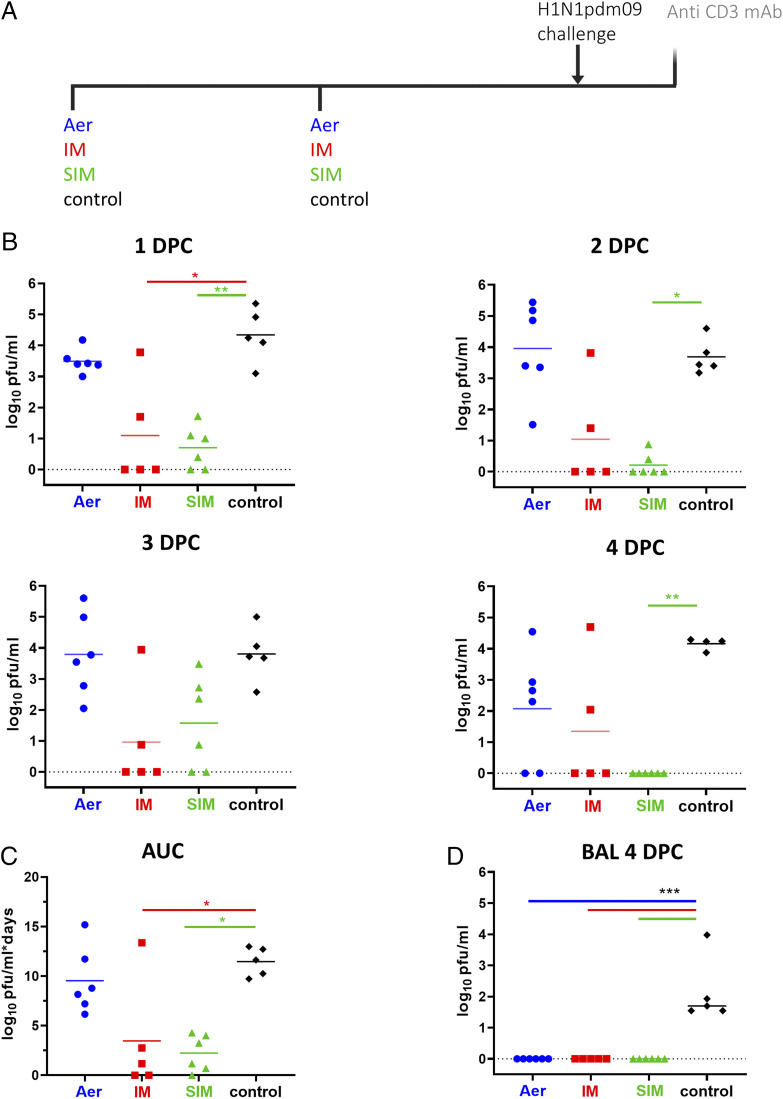
Experimental design and viral load in nasal swabs and BAL. (**A**) Babraham pigs were immunized with S-FLU by Aer, i.m., or simultaneously by Aer and i.m. (SIM) and boosted 3 wk apart. Control animals were left untreated. All animals were challenged with H1N1pdm09 virus 3 wk after the boost. Swabs were taken daily postchallenge, and all pigs were culled 4 DPC. Half of the pigs were infused intravenously with anti-porcine CD3 mAb 10 min prior to sacrifice. (**B**) Virus titer in nasal swabs measured by plaque assay at one, two, three, and 4 DPC. (**C**) Area under the curve (AUC) of viral titer in the nasal swabs over time. (**D**) Viral titer in the BAL 4 DPC. The data represent the average of two separate assays; each data points indicates an individual animal and the horizontal line the mean of the group. Data were analyzed using the Kruskal–Wallis test. Asterisks indicate a significant difference from the control group: *p < 0.05, **p < 0.01, ***p < 0.001.

To evaluate the efficacy of simultaneous pulmonary and systemic immunization, groups of six inbred Babraham pigs were immunized with S-FLU expressing NA and coated in the HA from H1N1pmd09 i.m. or by Aer alone or simultaneously by Aer and i.m. (SIM). The SIM group received the same total dose as the i.m. or Aer groups but split between the two sites. Untreated pigs were used as controls. The animals were boosted 3 wk later and, after a further 3 wk, challenged with H1N1pdm09 virus and culled 4 d after the challenge (Fig. 1A). Two pigs were culled before the end of the experiment because of underlying heart conditions, unrelated to the study, leaving five animals in the i.m. and control groups. Virus load was assessed in nasal swabs and BAL. The SIM pigs showed the greatest reduction of virus shedding in the nasal swabs at all time points except for the third DPC (Fig. 1B). In the i.m. group, two individuals shed virus consistently after challenge, but a significant reduction in viral load was achieved on 1 DPC. Aer immunization did not decrease virus shedding, although two pigs did not shed at 4 DPC (Fig. 1B). Overall i.m. and SIM significantly reduced the viral load in the nasal swabs over time, with an average area under the viral load/time curve of 3.46 and 2.23, respectively, compared with 9.53 and 11.46 of the Aer and control group (Fig. 1C). No virus was detected in BAL at 4 DPC in any of the immunized groups (Fig. 1D).

**FIGURE 2.**
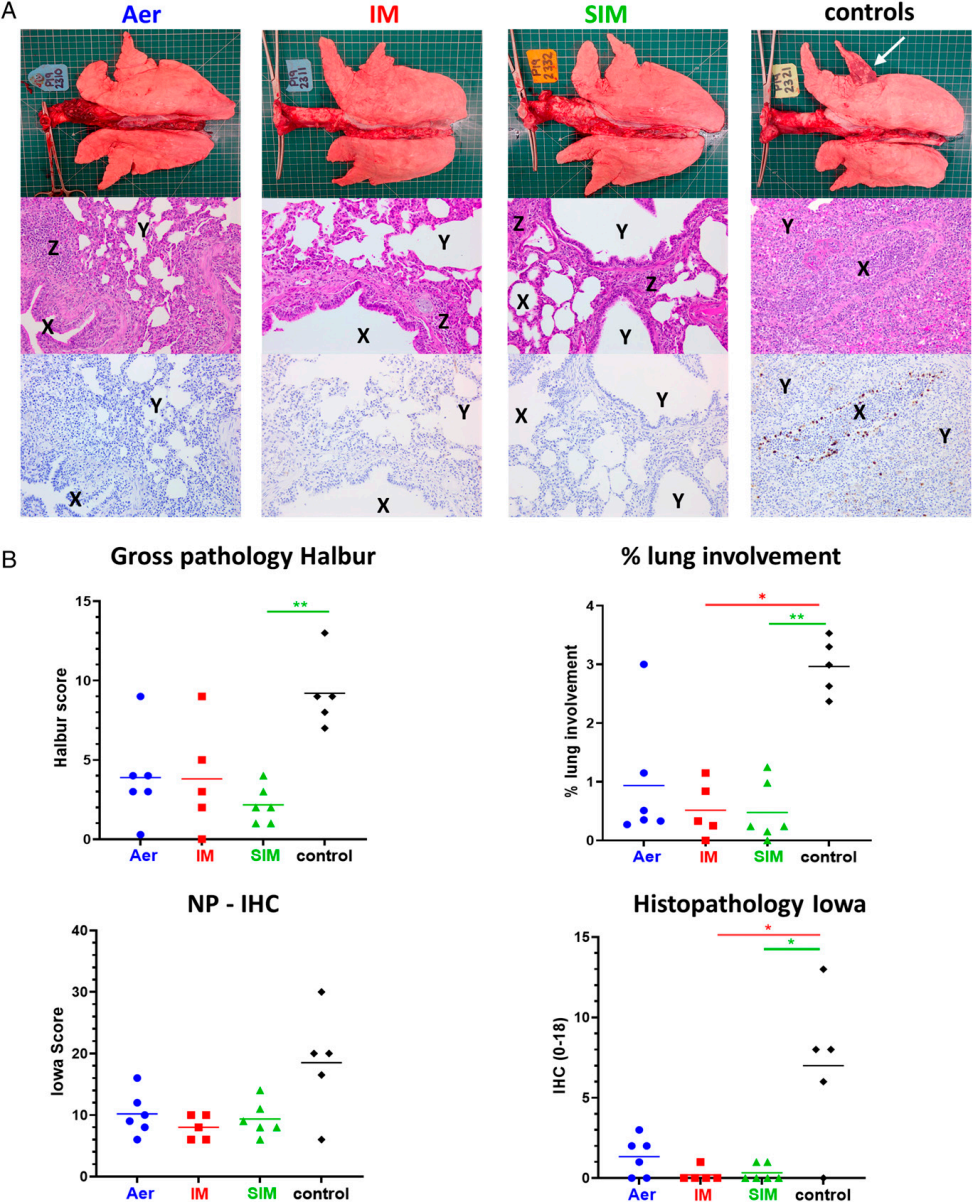
Lung pathology. Pigs were immunized with S-FLU by Aer, i.m., or simultaneously by Aer and i.m. (SIM), whereas control pigs were untreated. Three weeks postboost, pigs were challenged with H1N1pdm09. The animals were euthanized at 4 DPC, and lungs were scored for appearance of gross (indicated with a white arrow) and histopathological lesions. Representative gross pathology, histopathology (H&E staining; original magnification ×100), and immunohistochemical NP staining (original magnification ×200) for each group are shown (**A**). Mild lymphoplasmacytic infiltration in peribronchiolar areas and expansion of alveolar septa are present in groups Aer, i.m., and SIM (Z), whereas bronchioles (X) and alveolar spaces (Y) remain free of cellular exudation (white spaces). There is a marked exudation of inflammatory cells in bronchioli and alveoli in the control group (loss of white air spaces), and virus replication can be seen (brown labeling) in bronchiolar epithelial cells and inflammatory cells in bronchioli and alveoli. The gross and histopathological scores for each individual in a group and the group means are shown (**B**), including the percentage of lung surface with lesions, the lesion scores, and the histopathological scores (Iowa includes the NP staining). Pathology scores were analyzed using one-way nonparametric ANOVA with the Kruskal–Wallis test. Asterisks denote significant differences: *p < 0.05, **p < 0.01, compared with control.

The unimmunized animals showed typical gross pathology changes in the lungs with multifocal areas of consolidation in the cranial and medial lobes (Fig. 2A). A significant reduction in the extension and severity of the gross changes was observed in the i.m. and SIM groups (*p* = 0.02 and *p* = 0.005, respectively, compared with controls), with a trend toward improved pathology in the Aer group that did not reach statistical significance (Fig. 2B). A characteristic bronchointerstitial pneumonia, with bronchiolitis, alveolar exudation and lymphohistiocytic infiltration in the alveolar septa and peribronchial and perivascular areas was present in the unimmunized animals. A reduction in the severity of these changes was observed in the immunized groups (Fig. 2A). Labeling of influenza A NP by immunohistochemistry (NP-IHC) was seen in only one animal in the i.m. and two animals in the SIM groups (*p* = 0.02 and *p* = 0.03, respectively), whereas most nonimmunized pigs displayed abundant labeling. NP-IHC was reduced in the Aer group, although this was NS (*p* = 0.55). Despite a reduction in gross lesions score and number of virus-infected cells, no significant difference was found when histopathology and NP-IHC were combined in all immunized groups (Iowa score) (Fig. 2B).

These results indicate that i.m. and SIM immunization significantly reduced nasal virus shedding and pathology, with SIM being more effective in virus clearance and gross lung pathology reduction. Aer immunization did not significantly reduce nasal virus load or pathology. All immunization regimes eliminated virus in the BAL.

### Ab and B cell responses

**FIGURE 3.**
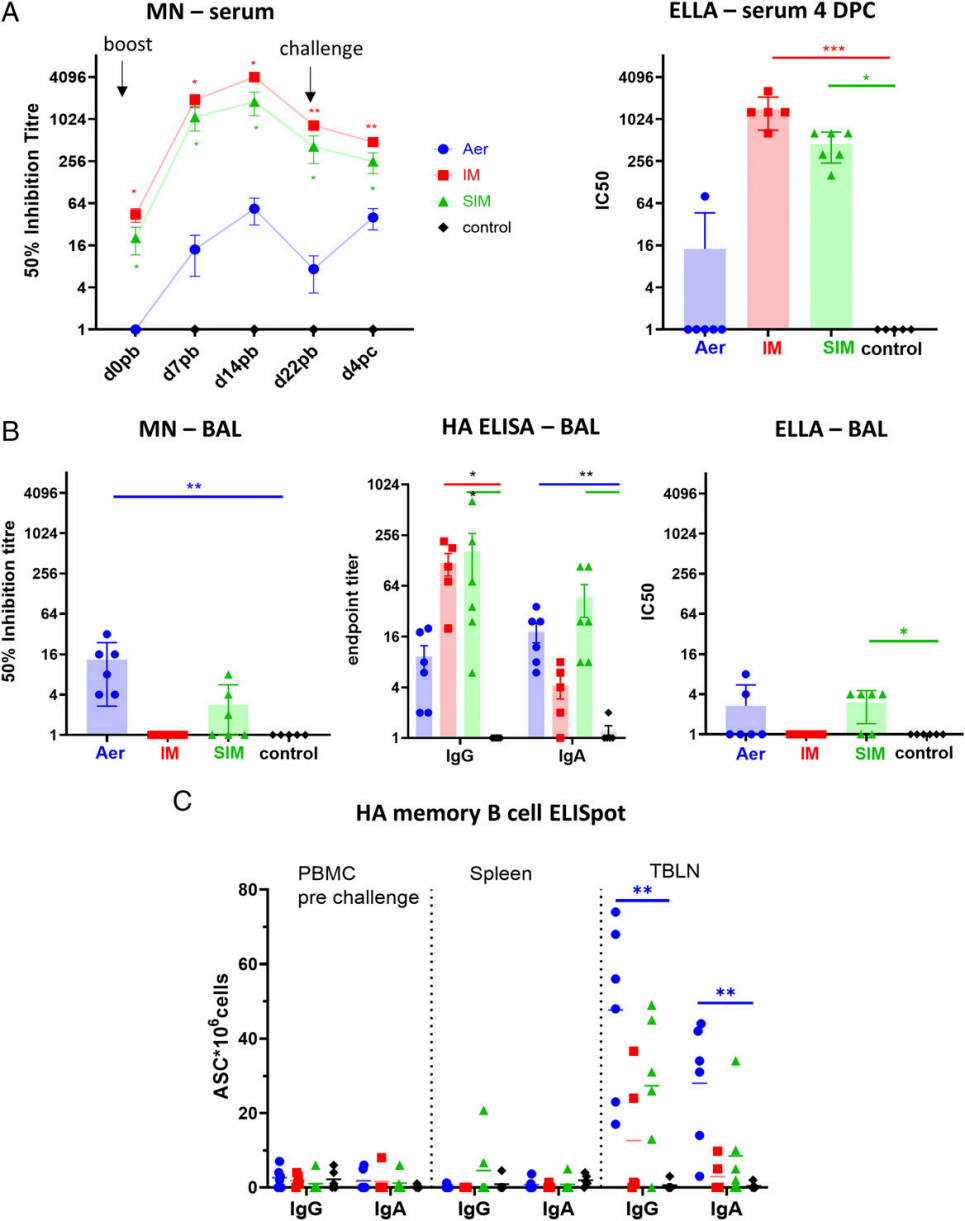
Systemic and local Ab responses. (**A**) Serum-neutralizing titers over time were determined by MN (in this study, shown as mean and SEM of two independent assays). NA inhibition activity was assessed by ELLA at 4 DPC. (**B**) BAL fluid was taken at 4 DPC, and virus neutralization was analyzed by MN, HA-specific IgG and IgA titers were measured by ELISA, and NA inhibition was assessed by ELLA. Each data point represents an individual animal. Each serum and BAL sample was assayed twice, and a mean computed. (**C**) HA-specific memory B cells were detected by ELISpot in PBMC (prechallenge), spleen, and TBLN 4 DPC. Each animal is represented by a symbol, and the mean is shown as a bar. Serum neutralization was analyzed with two-way ANOVA, whereas Kruskal–Wallis test was used for the analysis of NA neutralization in sera, BAL samples, and ELISpot data. Asterisks denote significance compared with control group (*p < 0.05, **p < 0.01, ***p < 0.001).

The serum-neutralizing titer against H1N1pdm09 in the i.m. and SIM groups increased after the boost to a peak of 4096 (50% inhibition titer) and 1812, respectively, at 14 DPB and declined by 22 DPB after the challenge (Fig. 3A). The Aer group had a much lower peak serum-neutralizing titer of 54 at 14 DPB (Fig. 3A). NA inhibition activity was assessed by ELLA at 4 DPC in the serum. i.m.-immunized animals showed the highest inhibition titer (1408) followed by SIM (453) and Aer (14.2) (Fig. 3A).

The neutralizing titer in the BAL was highest in the Aer group (13.4), and neutralizing activity was detectable in three of six SIM pigs (Fig. 3B). There was no detectable neutralization in the BAL of i.m.-immunized animals, although HA-specific Abs were present. High levels of anti-HA IgG were present in both i.m. and SIM groups (119.2 and 167, respectively), whereas the highest titers of IgA were detected in the Aer and SIM groups (18.3 and 46.6, respectively) (Fig. 3B). Only very low levels of NA inhibition were found in BAL compared with serum (Fig. 3B). We also evaluated the number of memory IgG and IgA HA-specific B cells in spleen, TBLN, and blood. Very few ASC were found in the blood before challenge or in spleen at 4 DPC (Fig. 3C). HA-specific, IgG-secreting ASC were detected in TBLN in the Aer (48 ASC/10^6^) and in the SIM (25 ASC/10^6^) groups. A lower number of HA-specific IgA ASC were present in these groups. Only two of five i.m.-immunized pigs had HA-specific IgG and IgA ASC in TBLN (Fig. 3C).

In summary, i.m. immunization with S-FLU induced high neutralizing Ab titers in serum but a limited response in BAL, although HA-specific Abs were present. Aer delivery generated the highest neutralizing titers in BAL but a very low serum response. The SIM group showed a high serum-neutralizing titer, although only half the magnitude of i.m. alone, whereas the BAL response was lower than in Aer only but still greater than i.m. Statistically significant numbers of HA-specific memory B cells were detected only in the Aer group in the local lung lymph nodes.

### Cytokine production by CD4 and CD8 cells in BAL

**FIGURE 4.**
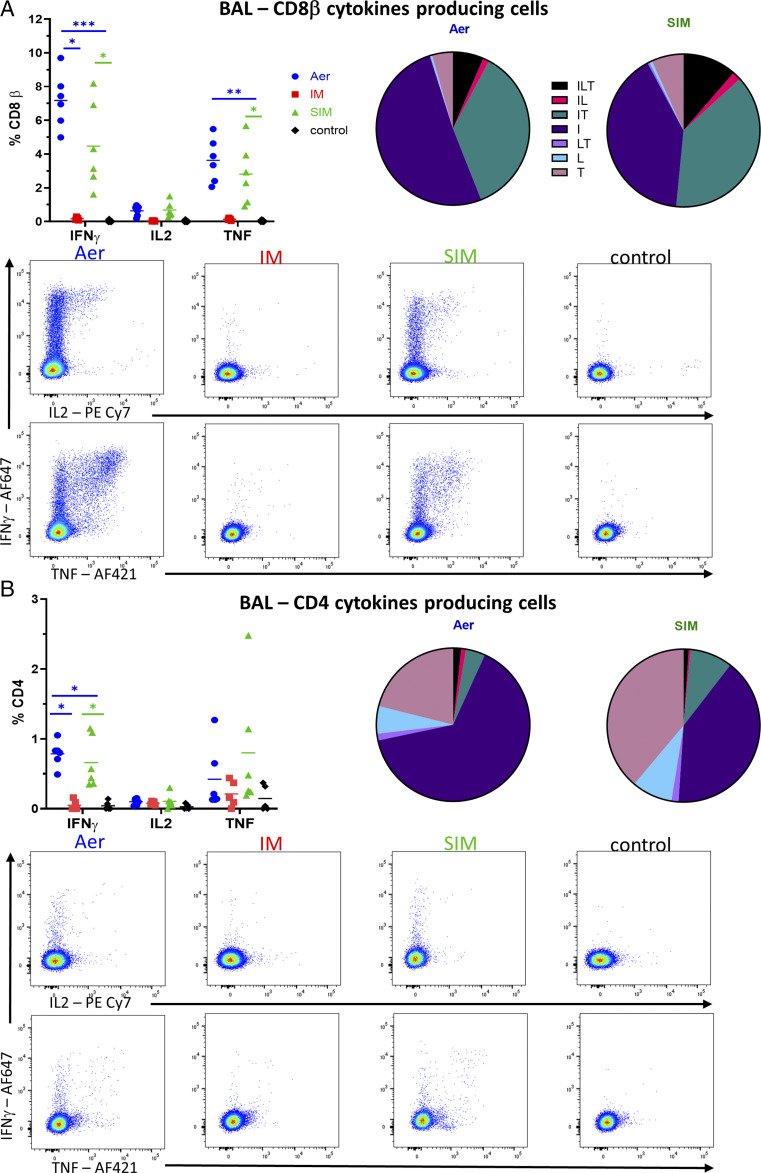
Cytokine secretion in BAL. BAL was collected at 4 DPC. Cryopreserved cells were thawed and stimulated with H1N1pdm09 (multiplicity of infection 1) and IFN-γ, and IL-2 and TNF cytokine secretion measured in CD8 (**A**) and CD4 (**B**) cells by intracellular staining. Each symbol represents an individual animal, and the mean is shown as a bar. The pie chart shows the mean proportion of single, double, and triple cytokine-secreting CD8 T cells for IFN-γ (I), TNF (T), and IL-2 (L). Representative plots show the cytokine-secreting cells within the CD8 (A) and CD4 (B) populations. Kruskal–Wallis test was used to compare responses between groups, and asterisks indicate significant differences (*p < 0.05, **p < 0.01, ***p < 0.001).

We analyzed cytokine production of BAL T cells by intracellular staining following ex vivo stimulation with H1N1pdm09. No T cell response was detected in the BAL of the i.m. group. In contrast, Aer and SIM immunization induced a strong T cell response. CD8 T cells in the Aer and SIM groups secreted mainly IFN-γ, followed by TNF, and the response in both groups was dominated by single IFN-γ producers (51%) followed by double secreting IFN-γ–TNF (36.2%) and a smaller proportion of triple secreting IFN-γ–TNF–IL-2 cells (6.5%) (Fig. 4A). The only significant CD4 responses were IFN-γ in Aer and SIM groups, and there were few double or triple cytokine-producing cells (Fig. 4B). Overall, Aer produced the strongest T cell response, dominated by IFN-γ–producing cells. SIM induced similar T cells functions, although the response was slightly lower in magnitude. i.m. delivery did not generate virus-specific T cells in the BAL at 4 DPC.

### NP-specific tetramer responses in the respiratory tract and blood

**FIGURE 5.**
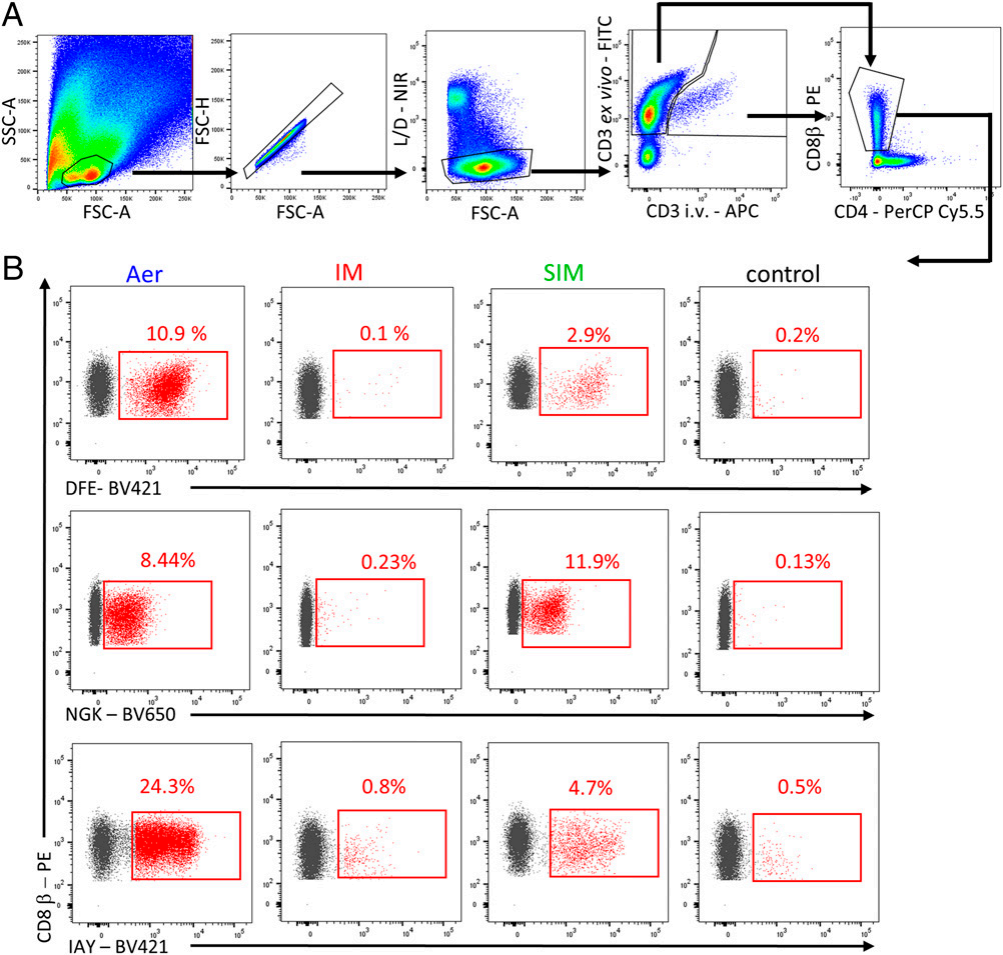
Tetramer staining. (**A**) Gating strategy for tetramer staining of BAL cells. (**B**) Representative FACS plots showing DFE, NGK, and IAY tetramers binding cells among the CD8 T cells in BAL in the different groups, highlighted in red (tetramer^+^ as percentage of CD8 β).

**FIGURE 6.**
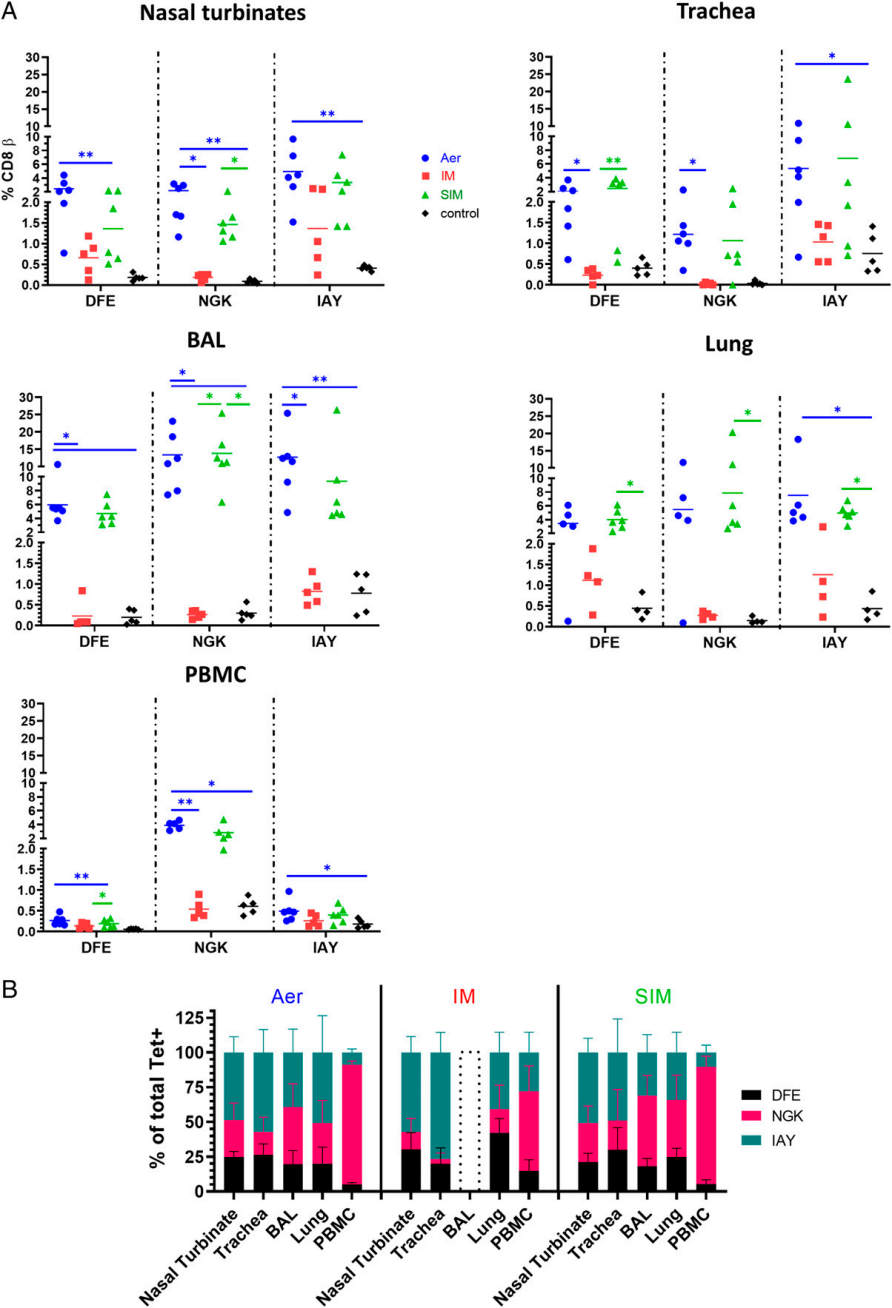
NP-specific tetramer responses in respiratory tissues and blood. (**A**) Percentages of DFE, NGK, and IAY tetramer^+^ CD8 T cells in the respiratory tract and PBMC. Each symbol represents an individual animal, and the mean is shown as a bar. (**B**) Proportion of each tetramer among total tetramers^+^ CD8 T cells in different tissues. A dotted histogram for BAL of the i.m. group indicates the absence of response. The data represent the average of two separate assays. Kruskal–Wallis test was used to compare responses between groups (A) and two-way ANOVA to compare the proportions of tetramers in the different tissues of each group of animals (B). Asterisks denote significant differences (*p < 0.05, **p < 0.01).

We enumerated S-FLU–specific CD8 T cells in blood and different parts of the respiratory tract (nasal turbinates, trachea, BAL, and lung) using three NP epitope tetramers: NP_290–298 DFEREGYSL_ (DFE), NP_101–109 NGKWMRELI_ (NGK), and NP_217–225 IAYERMCNI_ (IAY), as previously described (15) (Figs. 5, 6A, Table I). No responses were detected against the previously identified NP_252–260 EFEDLTFLA_ epitope in all immunized animals (data not shown).

**Table I.** List of Abs used for intracellular cytokine staining and NP tetramers enumeration

Ab	Clone	Isotype	Fluorophore	Supplier
Tetramer enumeration				
Anti-porcine CD3	PPT3	Mouse IgG1	FITC	Bio-Rad Laboratories
Anti-porcine CD4	74-12-4	Mouse IgG2b	PerCP Cy5.5	BD Biosciences
Anti-porcine CD8β	PG164A	Mouse IgG1	PE	Bio-Rad Laboratories
Anti-mouse IgG1	RMG1-1	N/A	Allophycocyanin	BioLegend
Intracellular cytokine staining				
Anti-porcine CD3	PPT3	Mouse IgG1	FITC	Bio-Rad Laboratories
Anti-porcine CD4	74-12-4	Mouse IgG2b	PerCP Cy5.5	BD Biosciences
Anti-porcine CD8β	PG164A	Mouse IgG1	PE	Bio-Rad Laboratories
Anti-porcine IFN-γ	P2G10	Mouse IgG1	Alexa Fluor 647	BD Biosciences
Anti-human TNF	Mab11	Mouse IgG1	BV421	BioLegend
Anti-porcine IL-2	A150D3F1	Mouse IgG2a	Unconjugated	Invitrogen
Anti-mouse IgG2a	RMG2a	N/A	PE Cy7	BioLegend
Anti-mouse IgG1	A85-1	N/A	BV650	BD Biosciences

In nasal turbinates, the response to IAY was the strongest (5% Aer, 1.4% i.m., and 3.4% SIM), followed by DFE (2.4% Aer, 0.7% i.m., and 1.4% SIM) and NGK (2.2% Aer, 0.2% i.m., and 1.5% SIM). The trachea showed similar specificity. The strongest response was detected in the BAL. NGK^+^ CD8 T cells were the biggest population (13.4% in Aer and 13.8% in SIM), followed by IAY^+^ (12.7% Aer and 9.4% SIM), and a lower response was found to DFE (6% Aer and 4.7% SIM). Strikingly, no tetramer staining was found in the BAL of the i.m. group, in agreement with the lack of intracellular cytokine staining (Fig. 4). In the lung, similar but lower tetramer-specific responses were detected for Aer (5.5% NGK, 7.5% IAY, and 3.5% DFE) and the SIM groups (7.9% NGK, 5% IAY, and 4.0% DFE) (Fig. 6A).

To evaluate the hierarchy of tetramer responses in different tissues, we calculated the proportion of each tetramer among total tetramer^+^ CD8 T cells (Fig. 6B). The proportions of IAY was much higher in all respiratory tissues compared with blood (*p* < 0.0001 for nasal turbinates compared with PBMC in the Aer group) (Fig. 6). The NGK response in blood was greater compared with all respiratory tissues (*p* < 0.0001 when nasal turbinates were compared with blood for the Aer group). In the i.m. group, less NGK^+^ cells were detected in all tissues compared with Aer and SIM. In particular, a significantly lower proportion of NGK^+^ CD8 T cells was found in i.m. PBMC compared with Aer (*p* = 0.01), although they were still the dominant NP specificity among CD8 T cells (57.1% of total tetramer^+^ CD8 T cells).

**FIGURE 7.**
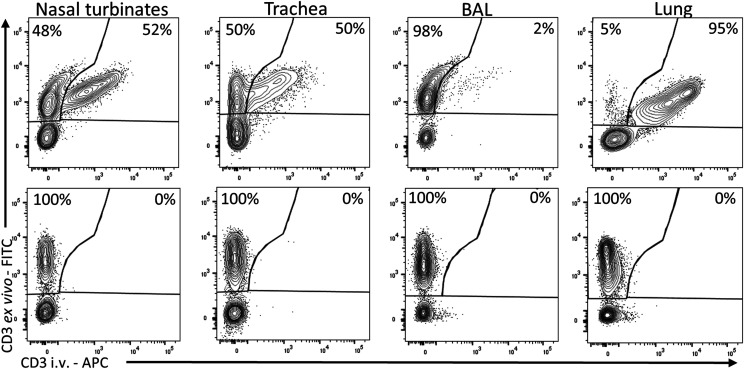
Porcine tissue-resident memory cells. Three pigs from the immunized groups and two control pigs were infused i.v. with CD3 mAb and culled 10 min later. Lymphocytes were isolated and stained ex vivo with the same clone of CD3 Ab labeled with FITC as described in the *Materials and Methods*. As the infused CD3 does not saturate all CD3 sites, some nasal turbinate, tracheal, and lung tissue T cells are double positive (intravascular cells). A proportion of BAL, nasal turbinate, and tracheal cells are unstained by intravascular mAb, indicating tissue residency (top row). The top row shows representative FACS plots for each tissue of infused animal. The bottom row shows staining in animals not infused with CD3 mAb and stained ex vivo. The reported proportion of CD3 ex vivo^+^CD3 i.v.^−^ and CD3 ex vivo^+^CD3 i.v.^+^ are calculated from total CD3^+^.

Finally, we assessed the numbers of TRM in the respiratory tissues by i.v. infusion of anti-porcine CD3 mAb as previously described (12) (Fig. 7). The majority of cells in the BAL were inaccessible to the mAb (82.4% average of all 11 animals treated with anti-CD3 mAb) and therefore tissue resident. In the nasal turbinates and trachea, 11.6 and 38.8% (average of all 11 animals) of single ex vivo–labeled CD3 cells (TRM) were detected, whereas in the lung, 95% of the T cells were double positive, perhaps reflecting known difficulties in extracting TRM and blood contamination (22). Tetramer-positive cells were detected in both TRM and blood-borne populations.

In summary, we detected strong NP tetramer-specific CD8 T cell responses in the nasal turbinates, trachea, BAL, and lung of Aer- and SIM-immunized animals. i.m. induced much lower number of tetramer-specific cells in all tissues and none in BAL. There was a different hierarchy of the response specificity in the respiratory tract compared with the blood, indicating that sampling blood does not represent responses in the local tissues.

## Discussion

In this study, we investigated different routes of immunization to determine the most effective in providing protection against influenza A virus in pigs. We tested the traditional systemic (i.m.) route used routinely in pigs and humans for influenza immunization and respiratory mucosal immunization, as used with the newer LAIV. We also performed SIM, previously shown to be highly protective, able to induce long-lasting immune response, and perhaps similar to prime and pull immunization regimes that have been investigated recently (5, 7, 8, 23). In our experiments, we administered S-FLU to the whole respiratory tract, a procedure that has been shown in mice to be superior to upper respiratory tract immunization in protecting against heterologous challenge (24). We were able to do so safely because S-FLU does not contain a viable RNA segment encoding HA. This obviates the two concerns that dictate restrictions of LAIV to the upper respiratory tract. First, the low level of replication of temperature-sensitive LAIV might cause lung pathology and, second, when used to protect against pandemic influenza viruses, reassortment of H5 or H7 with LAIV HA could occur (9, 11, 12, 25).

i.m. immunization induced a powerful neutralizing Ab response, and the viral load and lung pathology were both greatly reduced. However, T cell responses were weak in these animals and, strikingly, could not be detected in BAL at 4 DPC. Nor were neutralizing Abs present in BAL, although it should be noted that harvesting BAL involves considerable dilution, so low titers of Abs may be missed. These data indicate that i.m. immunization fails to develop lung responses, as previously reported in mice with seasonal human inactivated vaccine (26).

In contrast, in Aer animals, the serum-neutralizing titer was much lower, but neutralizing activity was detected in BAL. The viral load in nasal swabs was not reduced. There was a trend toward reduced gross and histopathology, although this did not reach significance. Aer animals made powerful CD8 and CD4 responses, detectable in BAL, a site containing almost exclusively TRM (12). Given the powerful T cell and neutralizing Ab responses in the BAL, it is surprising that Aer animals showed minimal reduction in nasal virus shedding and a weak effect on pathology, although there was no virus in the BAL. Similarly, in a previous experiment, pigs immunized by Aer with H3N2 S-FLU and challenged with heterologous H1N1pdm09 virus exhibited reduced lung pathology 5 DPC but no reduction in virus shedding (12). These data contrast with results in mice in which TRM have been shown both to protect against weight loss following heterologous influenza challenge and to reduce viral load (27–29). This suggests that T cell immunity in mice can both protect against clinical disease (weight loss) and reduce viral load, whereas in pigs, a powerful T cells response is insufficient to protect the upper respiratory tract from infection and shedding, although the lung viral load and pathology are reduced. Parallel experiments using the same immunization regimes and challenge virus in pigs and ferrets confirm that small animal models may not always predict the outcome in a large animal natural host, such as the pig (12). Correlative studies in humans suggest that cross-reactive T cells provide partial protection against influenza infection (30–32). Taken together, these data suggest that in large animals, pigs, and humans, cross-reactive T cell immunity can alleviate severe disease but not prevent upper respiratory infection.

The SIM animals showed a combination of the properties of the two immunizations. There was a good serum-neutralizing Ab response, and neutralizing Abs were also detected in BAL, but in contrast to i.m.-immunized animals, there was a powerful CD4 and CD8 T cell response in the BAL, lungs, and nasal turbinates. SIM animals showed greatly reduced viral load in nasal swabs and no detectable virus in BAL at 4 DPC together with reduced lung pathology. Our study shows that simultaneous systemic and Aer immunization may have the advantage of providing both protection against homologous challenge by induction of local and systemic neutralizing Abs and heterologous challenge, mediated by local and systemic T cells, including TRM. Systemic immunity may also be of benefit because influenza virus infection can also be systemic, and serum-neutralizing Abs may abolish viremia (33, 34). A heterologous challenge of SIM animals would be extremely interesting and confirm whether this immunization regimen is truly advantageous.

Simultaneous and prime pull immunization regimes have had mixed success. Although systemic prime followed by skin or reproductive tract pull successfully generated protective TRM in these tissues, it has been less easy to protect the respiratory tract by the same strategy (35, 36). Our earlier experiments with BCG in mice and cattle showed that simultaneous parenteral and intranasal administration of BCG provided improved protection, which we attributed to earlier local control of mycobacterial replication in the lungs postchallenge (5, 6). However, others have not replicated this in primates (37). Similarly, immunization with an intranasal lentiviral Tb vaccine containing Ag 85A (pull) after BCG prime failed to improve protection (38). In contrast, simultaneous immunization against influenza virus with an adenoviral vector in mice provided improved protection for up to 8 mo compared with s.c. or intranasal immunization alone, and TRM generated by this immunization regimen replicated in situ (7, 8). These differing outcomes may be partly explained by the need for additional signals as well as the Ag. For example, an adenoviral but not vesicular stomatitis virus vectored pull improved anti-Tb protection in mice (39), and an adenoviral vector encoding NP and 4-1BB ligand enhanced protection against influenza virus compared with NP alone (40). In the current study, i.m. S-FLU generated very weak T cell responses, and the data provide little evidence for recruitment of systemic T cells to the respiratory tract following infection (pull). However, i.m. S-FLU induce a strong neutralizing Ab response, an essential component of influenza vaccines, but the effectiveness of the SIM regimen might be improved by a systemic immunization able to generate a stronger circulating memory T cell reservoir, important for the replenishment of local responses (41).

Although local immune responses are important and, when combined with systemic responses, may provide optimal protection, it is fundamental to know how long they persist. Lung TRM have been shown to be short lived in mice, perhaps as a consequence of the high oxygen tension of the lung microenvironment (41, 42). However, experiments examining the persistence of influenza-specific memory indicate that a dividing lung memory population may persist for many months if Ag is retained in the lung (7). Our data using Ad85A vaccine showed that lung cells from mice immunized 23 wk previously could stimulate 85A-specific T cells to divide, indicating long-term persistence of Ag (23).

Our data illustrate another factor that may partly explain why some regimes have not worked. Although we have not analyzed the entire T cell repertoire in detail, the different hierarchy of specificities of tetramer^+^ CD8 T cells in i.m. compared with Aer animals suggests that the route of immunization can affect the T cell epitope specificity. Others have found that local cognate Ag recognition is fundamental for establishment of influenza-specific TRM (43, 44) and that local immunodominance is not always found in the circulating T cell pool, although it remains to be shown conclusively whether these differences in immunodominance affect protection (45).

Our results in this pig influenza challenge model indicate that SIM may offer advantages in protection against influenza viruses. SIM induces an excellent systemic Ab response, known to correlate with protection against homologous virus infection, as well as a powerful local TRM response, vital for protection against heterologous virus challenge. We suggest that development of SIM strategies for other respiratory pathogens, including SARS CoV-2, may be advantageous in providing both local protection and a high titer of Ab. SIM strategies should also take into account the need for local costimulatory signals and persistence of Ag in the lung.
